# Sanicula*Read before the San Francisco Medical Club.

**Published:** 1889-08

**Authors:** J. E. Lilienthal

**Affiliations:** San Francisco


### SANICULA.*

J. E. Lilienthal, M. D., San Francisco.

In the Congress of American Physicians and Surgeons, held at Washington during September, Dr. C. C. Rice, of New York, presented the report of the Committee on Mineral Springs. He states that over eight hundred letters were sent out to the different springs, and that the answers in the majority of cases were not alone unscientific, but also unsatisfactory.

He states, further, that nine-tenths of the mineral waters of the United States are still unanalyzed.

This is a sad commentary, and not without interest to us as homœopaths. The old school, who never miss an opportunity to have their fling at us at the smallness of the dose prescribed by members of our school, are staunch adherents of the benefits to be derived by a "cure" at one or the other springs, forgetting that the remedial agent for which that particular water may be prescribed contains perhaps but a fraction of a grain in a gallon.

It gives me pleasure to call your attention to a proving of the Sanicula Spring, Ottawa, Ill., by Dr. J. G. Gundlach, made from the water itself and potencies, and also one by Dr. Sherbino, of Texas, of potencies. If the symptoms derived from the provings be verified, and in part they have already been done, S. promises to be an antisporic of no mean value.

Our own State teems with mineral springs of undoubted value,

*Read before the San Francisco Medical Club.

and I trust the example set by these gentlemen will cause some of my hearers to emulate their example and give us provings which will result in benefiting not themselves alone but our cause as well.

Analysis, Prof. Silliman, Yale :*

| | |
|---|---|
| Sodium Chloride, | 92.7995 |
| Calcium Chloride, | 23.5699 |
| Magnesium Chloride, | 23.2687 |
| Sodium Bromide, | .3220 |
| Sodium Iodide, | .0826 |
| Lithium Bicarbonate, | trace |
| Sodium " | .9776 |
| Calcium " | 14.3494 |
| Ferrum " | .0979 |
| Potassium Sulphate, | 5.1246 |
| Calcium " | 9.6236 |
| Sodium Phosphate, | .0045 |
| Borax, | trace |
| Alumina, | .0117 |
| Silica, | .5394 |
| Organic matter, | trace |
| | 170.7734 |

| | |
|---|---|
| Carbon acid cub. in. at 60°.f | 25.6 |
| Density of water, | 1.0022 |

Lack of energy, with no stability of purpose; jumping from one work to another; never finishing what has been commenced. Depression of spirits, with feeling of some impending misfortune. Child stubborn and willful, gets angry and throws itself backward.

*Drosera.* Mental restlessness when reading; cannot dwell long on one subject; must change always to something else.

The depression, with feeling of impending misfortune, we find marked under *Calc-c.*, as well as the stubbornness and irritability, the latter also recalling the mental symptoms in childhood of Cham. and Cina.

Like Borax it has < from downward motion. Not alone was this symptom developed in a child, but the doctor who could not endure the downward motion of elevator, has been cured of this failing since making his proving.

* The water is without odor or color, and of an agreeable and slightly saline taste.

Dull headache, which seems to be felt in the morning; gets worse about noon, and is better toward evening. The pains are < from motion, leaning the head forward, any draught or noise. Better from leaning head back, cool, open air and wrapping head up warm, rest and sleep.

Great accumulation of dandruff was noted in all, the hair becoming dry and lustreless, in this reminding us of Alum and Kali-c., but it has the itching more marked when the head gets warm.

Like Calc. the child sweats profusely about the head and neck during sleep, wets the pillow all around.

In scrofulous diseases of the eye, especially in severe cases of blepharitis, S. promises to be useful, its action being similar to Graphite, etc., having the same sticky discharge, which dries, forming white scales, and having an ulcerated surface under the scabs. Lids agglutinated in morning. Upon the nasal mucous membrane we find it producing a profuse, thick, acrid discharge, which, as it becomes thicker forms into scabs and clinkers, which are thrown off both from the anterior and posterior nares. The character of this discharge, with its tendency to form clinkers, reminds us strongly of Kali-bich., but it differs in its concomitants, as the dryness of the nose at night with the < of the discharge in-doors, and in the mornings bears more resemblance to the < of Nux.

Dr. Sherbino reports a clinical symptom similar to what we find under Squills. "On awakening the child rubs its nose and eyes with the hand." Mouth dry, yet still no desire for drink; apthæ on tongue and inside of lips and cheeks, with foul breath. Appetite is improved, but food is not assimilated, as the patient emaciates; the digestion is slow, and we have bloating after eating, causing person to open clothes; sour, acid eructations. Nausea coming on while eating, with vomiting of the food; in children the vomited matter is in large tough curds. The vomiting of large curds with falling off in a sleep is very similar to Æthusa, but the Sanicula condition is a later and graver condition. Emaciation, especially about the neck, more pronounced, and the stools different. It may be watery, as sometimes the Æthusa stools are, but it is oftener stools of lumps of curds, smelling like rotten cheese. It need not always be this character, in fact, the stools are apt to be changeable, at times resembling the Magn-carb. condition, at other times more like Rheum, having the characteristic of turning green after standing, but lacking the sour smell which accompanies the Rheum stool.

The effect on the stools seems primarily to cause an increase in quantity and to cause the stools to become softer, this is followed by a paretic condition of the lower bowel, with inability to expel its contents, immaterial if they be soft or large, hard and dry, or first part hard, second natural in consistency.

During stool intense straining, with tendency for the stool to slip back on stopping to catch his breath. This intense straining, to the extent of even grasping the seat, finds its only resemblance under Alumina, but the slipping back of the stool when having been partly expelled has its counterpart in Silicea and Magn-mur. All have the straining, even with soft stools, but the pains with all three are more in anus and rectum, while under Sanicula the perineum not alone pains during but continues to feel sore and burn several hours after stool. (Lyc. contractive pain in perineum for many hours after hard, scanty stool.)

The urine is increased in quantity, frequently obliged to rise during the night.

Menstrual flow irregular in time, but seems to be increased in quantity, and clinically has been prescribed successfully for menorrhagia. It needs further proving in this direction. Leucorrhœa smelling like strong fish-brine; this odor seems to be peculiar to the remedy, as the male prover had the same odor about the glands after intercourse. Cough caused by a tickling under sternum, with so much soreness of the chest that he holds the chest with hands; < in morning and in warm room and from laughing and speaking, and > in open air, with considerable rattling in the chest and expectoration of yellow, mattery lumps.

The symptoms of the back remind us strongly of Rhus as far as the concomitants are concerned. We have some amelioration from motion, also by pressing against some hard substance and by lying on the back. It is a tired, weak feeling, more in the lumbo-sacral region, coming on in the morning after rising, increasing in intensity until noon, gradually passing away in the course of the afternoon.

The symptoms of the extremities require further proving, but we have some symptoms reminding us of Calc. and Sulph. The hands and feet are cold and clammy, with considerable foul, fetid perspiration between the toes. We find, on the other hand, a contrary condition. Heat of the palms and soles, so that the prover sticks out the feet to cool them.

I do not wish to weary you with a full synopsis of the symptoms, which you can read for yourselves. If I have succeeded in wakening in you any interest for the drug, or for examining the springs of our own State, my purpose has been attained. It has been used clinically in enough cases of inanition to prove its value; and certainly the emaciation, the disturbed abdomen, the perspiration about the head and neck, all symptoms which we have all met with so often in these conditions, are indications enough to lead us to make a closer study of this natural spring in such cases of marasmus that we may be called upon to treat.

